# Compartment-Specific Differences in the Activation of Monocyte Subpopulations Are Not Affected by Nitric Oxide and Glucocorticoid Treatment in a Model of Resuscitated Porcine Endotoxemic Shock

**DOI:** 10.3390/jcm11092641

**Published:** 2022-05-08

**Authors:** Tomasz Skirecki, Barbara Adamik, Claes Frostell, Urszula Pasławska, Stanisław Zieliński, Natalia Glatzel-Plucińska, Mateusz Olbromski, Piotr Dzięgiel, Waldemar Gozdzik

**Affiliations:** 1 Laboratory of Flow Cytometry, Centre of Postgraduate Medical Education Marymoncka 99/103, 01-813 Warsaw, Poland; 2 Clinical Department of the Anaesthesiology and Intensive Therapy, Wroclaw Medical University, 50-367 Wroclaw, Poland; barbara.adamik@umw.edu.pl (B.A.); stanislaw.zielinski@umw.edu.pl (S.Z.); waldemar.gozdzik@umw.edu.pl (W.G.); 3 Department of Anaesthesia and Intensive Care, Karolinska Institutet, Danderyd Hospital, 182 57 Stockholm, Sweden; claes.frostell@ki.se; 4 Institute of Veterinary Medicine, Faculty of Biological and Veterinary Sciences, Nicolaus Copernicus University in Torun, 87-100 Torun, Poland; urszula.paslawska@umk.pl; 5 Department of Internal Medicine and Clinic for Horses, Dogs and Cats, Wroclaw University of Environmental and Life Sciences, 50-375 Wroclaw, Poland; 6 Department of Histology and Embryology, Department of Human Morphology and Embryology, Wroclaw Medical University, 50-367 Wroclaw, Poland; natalia.glatzel-plucinska@umw.edu.pl (N.G.-P.); mateusz.olbromski@umw.edu.pl (M.O.); piotr.dziegiel@umw.edu.pl (P.D.); 7 Department of Physiotherapy, University School of Physical Education, 51-612 Wroclaw, Poland

**Keywords:** sepsis, porcine model, compartmentalization, innate immunity, shock, monocyte

## Abstract

Inhaled nitric oxide (iNO) remains one of the treatment modalities in shock, and in addition to its vasoactive properties, iNO exerts immunomodulatory effects. We used a porcine model of endotoxemia with shock resuscitation (control) and additional treatment with iNO and a steroid (treatment group). After 20 h, bone marrow (BM), peripheral blood (PB), and bronchoalveolar lavage fluid (BALF) were collected to analyze the immunophenotype and mitochondrial membrane potential (Δφ) in three subsets of monocytes. In both groups, SLA-DR expression decreased twofold on the circulating CD14^+^CD163^+^ and CD14^−^CD163^+^ monocytes, while it did not change on the CD14^+^CD163^+^. Δφ increased only in the CD14^−^CD163^+^ subpopulation (0.8 vs. 2.0, *p* < 0.001). The analysis of compartment-specific alterations showed that nearly 100% of BALF CD14^+^CD163^+^ and CD14^−^CD163^+^ monocytes expressed SLA-DR, and it was higher compared to PB (32% and 20%, *p* < 0.0001) and BM (93% and 67%, *p* < 0.001, respectively) counterparts. BALF CD14^+^CD163^+^ had a threefold higher Δφ than PB and BM monocytes, while the Δφ of the other subsets was highest in PB monocytes. We confirmed the compartmentalization of the monocyte response during endotoxemic shock, which highlights the importance of studying tissue-resident cells in addition to their circulating counterparts. The iNO/steroid treatment did not further impair monocyte fitness.

## 1. Introduction

Sepsis was designated a major global health priority by the WHO in 2017 [1]. Despite more than 30 years of intensive preclinical and clinical research, no effective sepsis treatment has been introduced into clinical practice [2]. The current guidelines are limited to antimicrobial and fluid therapies in addition to the intensive care, and no specific immunomodulatory treatments are recommended, as those that were tested failed to improve patients’ outcome [3]. The failure to successfully translate any of the numerous promising preclinical therapies can be attributed to the use of simplified small animal models without intensive care treatment and often irrelevant study protocols, e.g., pre-treatment regimens [2,4].

Sepsis, which is defined as a dysregulated host response to infection [5], is related to multiple organ failure with the cardiovascular system, lungs, and kidneys being the most commonly affected [6]. The pathophysiology of acute organ failure in sepsis is extremely complex and is related to the activation of tissue macrophages and the release of inflammatory mediators [2]. On the other hand, functional monocyte/macrophage populations are necessary to combat the underlying infection and prevent secondary infections [7]. Therefore, potential immunomodulatory treatment should reduce inflammatory organ injury while preserving the viability and competence of the immune cells. Inhaled nitric oxide (iNO) has been proposed as an organ-protective treatment that also has vasoactive action [8]. iNO has also been shown to modulate systemic inflammation by reducing the production of inflammatory mediators and adhesion molecule expression at the tissue level [8]. iNO was shown to downregulate the lung cells NF-κB activation and the production of IL-6 and MCP-1 leading to decreased influx of neutrophils and monocytes [9,10]. Interestingly, NO up-regulates the expression of the glucocorticosteroid receptor, which also mediates the immunomodulatory effects of steroids [11]. As a low dose of steroids, hydrocortisone is recommended in the treatment of septic shock with the ongoing requirement for vasopressor therapy [3]; the combination of these two compounds is of special interest. In a porcine model of endotoxemia, the use of hydrocortisone together with iNO reduced NF-κB and TNF and tissue injury in the lungs, kidney, and liver [11]. We have previously shown that only the combination of iNO and hydrocortisone was able to reduce the degree of acute kidney injury in a porcine endotoxemia model and ischemia-reperfusion model [12,13]. In the latter, the combined treatment reduced the expression of the endotoxin receptor TLR4 in the kidneys. Unfortunately, the cited studies analyzed the effects of iNO on the tissue level and little is known about the effects of the exogenous NO on the monocytes and macrophages in sepsis. The immunomodulatory effects of hydrocortisone alone are widely described and include the depletion of circulating CD14^+^CD16^+^ intermediate monocytes and reduction in monocytic expression of IL-10 and CD163 in healthy volunteers [14]. In septic patients, less is known about the exact effects of glucocorticosteroids on monocytes, but hydrocortisone was shown to reduce monocytic HLA-DR expression but increased phagocytic capacities [15,16]. Taken together, these observations prompted us to investigate the effects of combined iNO and steroid treatment on monocyte and macrophage activity during endotoxemia.

This study aimed to further assess the effects of iNO + hydrocortisone treatment on monocytes in a model of resuscitated porcine endotoxemia. We used this model due to its high translational value and the opportunity to perform both physiological and immune studies in the same animals, which is in accordance with the 3Rs rule (*Replacement*, *Reduction*, and *Refinement*).

## 2. Methods and Materials

### 2.1. Animals

All animals were housed and handled according to the Guide for the Care and Use of Laboratory Animals (EU Directive 2010/63/EU for animal experiments). Experiments were conducted at the Department of Internal Medicine and Clinic of Diseases of Horses, Dogs and Cats at Wroclaw University of Environmental and Life Sciences. All procedures were performed by intensive care specialists under the supervision of a veterinarian. Animal experiments were performed upon obtaining the consent of the Local Ethics Committee in Wroclaw, Poland.

### 2.2. Endotoxemia Model

The study was performed on healthy, 4-month-old domestic pigs of mean weight 27 kg. Animals were fasted overnight with access to water. Each pig underwent anesthesia, instrumentation, and catheterization. Before instrumentation, animals were allocated into two groups, using quasi-randomization based on the order of transfer from animal facility, as follows:

1. Treatment group: standard treatment + inhaled NO and IV hydrocortisone;
2. Control group: standard treatment.

**Figure 1:**
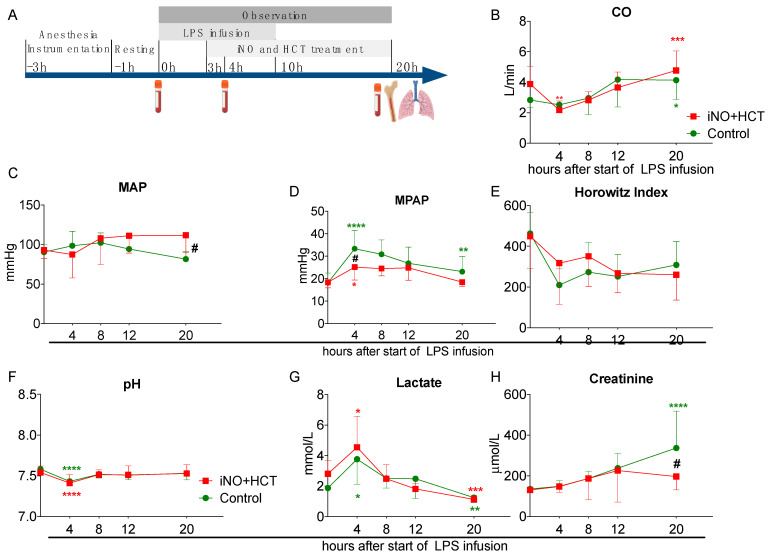
Model of porcine-resuscitated endotoxemic shock and intensive care treatment. (**A**) Schematic presentation of the study protocol. Kinetic changes in the clinical parameters over the course of the endotoxemia model: (**B**) Cardiac output (CO), (**C**) Mean arterial pressure (MAP), (**D**) Mean pulmonary arterial pressure (MPAP), (**E**) Horowitz Index, (**F**) pH, (**G**) Serum lactate concentration, and (**H**) Serum creatinine levels. Mean values ± SD are presented. Baseline, T4 h T8 h n = 9/group, T20 h n = 6/group. The statistical significance of differences between values at baseline and given timepoints for each group: * *p* < 0.05, ** *p* < 0.01, *** *p* < 0.001, **** *p* < 0.0001. The statistical significance of the differences between groups at given timepoints: # *p* < 0.05. LPS lipopolysaccharide, HCT hydrocortisone.

Shock was induced in all animals by endotoxin infusion for a total of 10 h. To induce shock, each pig received an IV infusion of endotoxin (Lipopolysaccharide, LPS) of *Escherichia coli* O111:B4 (SIGMA, Gothenburg, Sweden, Chemical lot 110K41 10) mixed in sterile water; the study procedures were previously described in detail [17]. Briefly, after baseline measurements, each animal received an initial dose of LPS (2.5 µg kg^−1^ h^−1^) intravenously over a period of 90 min, and then the dose was lowered (0.5 µg kg^−1^ h^−1^) for the remaining 8.5 h (a total of 10 h). We then followed all the animals for another 10 h with no endotoxin administered (Figure 1A). Fluid and vasopressor support were administered in a protocolized manner. Throughout these experiments, the animals received a constant IV fluid infusion of 0.9% saline/5% glucose (1:1) at 15 mL kg^−1^ h^−1^. During the experiment, the animals received IV boluses of saline, if the mean arterial pressure (MAP) was less than 60 mmHg and the pulmonary capillary wedge pressure (PCWP) was less than 6 mmHg, in order to reach a PCWP of 8 mmHg. If the PCWP was above 8 mmHg and the MAP was less than 60 mmHg, an IV infusion of norepinephrine of 0.025 μg/kg^−1^ min^−1^ was started and titrated until a MAP above 60 mmHg was achieved. The maximal norepinephrine dose was 0.6 μg/kg^−1^ min^−1^. If the MAP was maintained above 60 mmHg, norepinephrine was slowly phased out if possible. All animals received an antibiotic, cefuroxime (GlaxoSmithKline, Solna, Sweden) 750 mg IV before instrumentation and the dose was repeated every 8 h. The antibiotic was used as a prophylaxis of surgical infection and to mimic the clinical practice [4]. Body temperature was monitored with a PAC thermistor in order to keep the animals normothermic (37 °C–38 °C) using heating blankets or external cooling if needed. After 20 h of the experiment, the animals were killed by intravenously administering sodium pentobarbital (Morbital, Biowet, Pulawy, Poland) 133.3 mg/mL, 0.6 mL/kg^−1^.

Anesthetic management was recently described elsewhere [12]. Briefly, the induction of anesthesia was performed with an intramuscular injection of zolazepam/tiletamine 4 mg kg^−1^ dissolved in medetomidine of 0.08 mg kg^−1^. The trachea was intubated, and the piglets were ventilated in a pressure-controlled mode using a Servo 900C ventilator (Siemens-Elema AB, Solna, Sweden) at an inspired fraction of oxygen (FiO_2_) of 0.3 and a positive end-expiratory pressure (PEEP) of 5 cm of H_2_O. The inspiratory pressure was set to keep the piglet normo-ventilated. Anesthesia was maintained with propofol (3–6 mg kg^−1^ h^−1^ iv; Fresenius Kabi Poland, Warsaw, Poland) and fentanyl (0.8–1.3 μg kg^−1^ h^−1^ iv; Polfa, Warsaw, Poland). Anesthetic doses were increased during instrumentation and then lowered to standard sedation doses for the remaining study period. The instrumentation consisted of a carotid arterial line, a central venous catheter, a pulmonary artery catheter, a peripheral IV catheter in an ear vein, and a urinary catheter through a mini-laparotomy. The adequacy of anesthesia during maintenance was assessed based on hemodynamic responses and additional IV fentanyl (25–50 µg) doses were given if the animals were judged to be inadequately anesthetized. The surgery was performed by the same operators. Physiological data were analyzed at baseline, 4 h, 8 h, 12 h, and 20 h after the start of the LPS infusion by the same researchers. Researchers could not be blinded to the experimental group, due to the need to deliver and control nitric oxide to the treated group. The ARRIVE reporting checklist is presented in Supplementary Table S1.

### 2.3. Sampling

Peripheral blood samples were collected from the carotid artery catheter according to the study protocol into tubes containing EDTA. Bronchoalveolar lavage was performed on autopsy from excised lungs by inserting a silicon catheter via the trachea until it was wedged in the periphery. Then, the lungs were lavaged five times with 20 mL of normal saline. The bronchoalveolar lavage fluid (BALF) was pooled, filtered via sterile gauze, and centrifuged at 300× *g*. The cellular pellet was resuspended in 0.5 mL of PBS. Bone marrow was aspirated from the femurs using a bone biopsy needle and filtered. All samples were collected within 30 min of the animal’s death and immediately transferred to the laboratory to perform flow cytometry assays.

### 2.4. Clinical Chemistry

Blood samples for hematological, biochemical, and arterial blood gas analysis (including measurements of the lactate concentration) were drawn at baseline and at 4, 8, 12, and 20 h of the experiment. Serum lactate was measured using a Konelab PRIME 30i analyzer (Thermo Scientific, Waltham, MA, USA) and hematological parameters were measured with the Animal Blood Counter Vet (Horiba Medical, Montpellier, France)

### 2.5. Flow Cytometry

Red blood cells were lysed in all samples with an ACK Lysing Buffer (Thermo Fisher Scientific, Waltham, MA, USA). After centrifugation (300× *g*, 5 min), the cells were resuspended in PBS. The following antibodies for immunophenotyping of the monocytes were used: anti-CD14-PE.Cy7 (clone M5E2, BioLegend, San Diego, CA, USA), anti-CD163 (2A10/11, Bio-Rad (AbD Serotec), Hercules, CA, USA) labeled with Zenon AlexaFluor 647 mouse IgG1 (Thermofisher), and anti-SLA-DR-FITC (2E9/13, Bio-Rad (AbD Serotec)). Staining was performed for 30 min at room temperature and cells were washed with PBS and resuspended in 0.5% formaldehyde in PBS. For the analysis of the mitochondrial membrane potential, cells were stained with anti-CD14 and anti-CD163 as described above, and then stained with JC-1 dye (Thermofisher) according to the manufacturer’s protocol. Cells were analyzed using the FACSCanto II flow cytometer and FACSDiva software (BD Bioscience, San Jose, CA, USA). A minimum of 20,000 mononuclear cells were recorded and analyzed in FlowJo 10.0 software (FlowJo LLC, Ashland, OR, USA).

### 2.6. Statistical Analysis

The sample size was decided based on the previous study [13], but it was not calculated, due to the lack of data on the analyzed monocytic measures in pigs. Data are expressed as means and standard deviations (SD). The normality of the data was checked with the Shapiro–Wilk test. Multiple comparisons were performed using a 2-way ANOVA with Tukey’s multiple comparison test. Data were analyzed and graphically depictured using the GraphPad Prism 7 (GraphPad, Inc., San Diego, CA, USA) software.

## 3. Results

### 3.1. Induction of Shock by Prolonged Lipopolysaccharide Infusion

A total of 18 animals were enrolled in the study, 9 in Group 1 (the treatment group; 3 females, 6 males) and 9 in Group 2 (the control group; 4 females, 5 males). The gender composition of the groups was balanced (*p* = 0.62, chi-square test). Data from all animals were analyzed. After completing the instrumentation and resting phase, all animals were alive and hemodynamic parameters were within physiological ranges. The mortality within the 20-h observation period in both groups was equal (3/9 pigs died in each) and all deaths occurred at 12-20 h after induction of shock. Four hours after the start of the infusion of endotoxin, a decrease in the cardiac output (CO) was observed in both groups (control: 2.84 L/min vs. 2.52 L/min; treatment: 3.88 L/min vs. 2.18 L/min, *p* < 0.01) and reached significance in the treatment group (Figure 1B). At 20 h after the start of endotoxemia, the CO increased to the normal value in both groups (control: 4.13 L/min; treatment: 4.76 L/min; Figure 1B). The arterial mean blood pressure (MAP) was maintained throughout the observation period within the normal range, but at the 20-h timepoint, it was significantly higher in the treatment group than in the control group (111.8 mmHg vs. 81.7 mmHg, *p* < 0.05, respectively; Figure 1C). In both groups, a rapid increase in the mean pulmonary artery pressure (MPAP) was evident, but after 4 h of LPS infusion, it was higher in the control than the treated group (33.3 mmHg vs. 25.14 mmHg, *p* < 0.05) and remained significantly elevated even after 20 h (23.1 mmHg vs. 18 mmHg at baseline, *p* < 0.01; Figure 1D). Endotoxin infusion induced rapid impairment of the gas exchange, as evidenced by the drop in the Horowitz Index below 300, which indicates mild acute respiratory distress syndrome, and there were no differences between the experimental groups (Figure 1E). Endotoxemic shock was evident by a drop in the arterial blood pH (control: 7.58 to 7.53, *p* < 0.001; treated: 7.54 to 7.41, *p* < 0.001) and an increase in the lactate concentration (control: 1.87 mmol/L to 3.77 mmol/L, *p* < 0.05; treated: 2.80 mmol/L to 4.55 mmol/L, *p* < 0.05; Figure 1F,G). The baseline pH and lactate concentration were assessed after 1 h of anesthesia, before the onset of endotoxin infusion, and their values were similar to those previously reported [17,18]. Interestingly, the serum creatinine concentration increased significantly after 20 h only in the control group, while it remained lower in the treatment group (336.7 µmol/L vs. 196.5 µmol/L, *p* < 0.05, respectively; Figure 1H). Altogether, we confirmed the induction of endotoxemic shock in our model of LPS infusion and resuscitation, in which the combined iNO plus hydrocortisone treatment improved MAP, MPAP, and kidney function.

### 3.2. Endotoxemic Shock Induced Rapid Changes in the Profile of Circulating Monocytes

**Figure 2:**
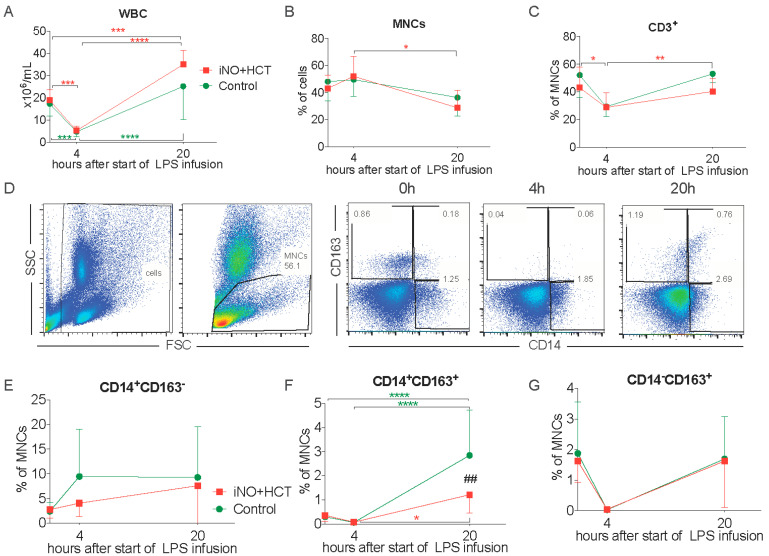
Endotoxin-induced changes in the circulating cell counts and phenotypes. Time-course changes in: (**A**) White blood count (WBC), (**B**) Circulating mononuclear cells (MNCs), and (**C**) CD3^+^ T-cells. (**D**) Representative dot plots of the flow cytometric analysis of blood monocytes; first, all cells were gated, and then the gate for the MNCs was applied and this population was analyzed for the expression of CD163 and CD14 before the infusion of endotoxin, 4 h and 20 h after the start of endotoxemia. The frequency of: (**E**) CD14^+^CD163^−^ monocytes, (**F**) CD14^+^CD163^+^ monocytes, and (**G**) CD14^−^CD163^+^ monocytes is shown. Mean values ± SD are presented. Baseline, T4 h T8 h n = 9/group, T20 h n = 6/group. The statistical significance of differences between values at different timepoints for each group: * *p* < 0.05, ** *p* < 0.01, *** *p* < 0.001, **** *p* < 0.0001. The statistical significance of differences between groups at given timepoints: ## *p* < 0.01. FSC forward scatter, SSC side scatter.

Serial blood sampling enabled us to analyze the changes in the circulating leukocytes triggered by the endotoxemia. LPS infusion induced a rapid drop in the total white blood count with a subsequent rise to baseline levels in the control group and even leukocytosis in the treatment group (Figure 2A). The frequency of mononuclear cells was significantly lower at 20 h in comparison to 4 h of LPS instillation in the treatment group (Figure 2B). There was also a rapid decrease in the CD3^+^ T cell count after 4 h of endotoxemia, which rebounded at 20 h, and these changes reached significance in the iNO+ hydrocortisone group (Figure 2C). We focused on the changes in the circulating monocytes. For this purpose, we used a combination of CD14 and CD163 markers, which was shown to reliably distinguish porcine monocyte subsets into: CD14^+^CD163^−^, CD14^+^CD163^+^, and CD14^−^CD163^+^ cells [19,20] (Figure 2D). LPS infusion insignificantly increased the fraction of circulating CD14^+^CD163^−^ monocytes, resembling human classical CD14^hi^ monocytes (Figure 2E). Interestingly, although we observed an expansion of the rare CD14^+^CD163^+^ cells in both groups, it was significantly higher in the control animals (Figure 2F). CD14^−^CD163^+^ monocytes, which are equivalent to human intermediate monocytes [19], showed high variability, and therefore, we did not observe statistically significant changes in this population (Figure 2G). Taken together, these observations indicate rapid leukopenia after the onset of LPS infusion, which was followed by an increase in the polymorphonuclear cells and expansion of the CD14^+^CD163^+^ monocytes, especially in the control animals.

### 3.3. Activation Status of Circulating Monocytes during Endotoxemic Shock

**Figure 3:**
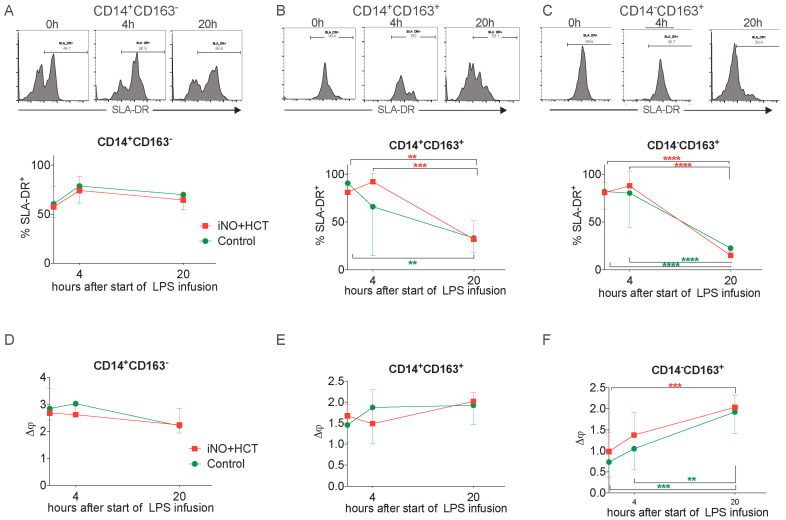
Effects of endotoxemia on the activation status of circulating monocytes. The kinetics of SLA-DR expression on subpopulations of circulating blood monocytes with representative histograms from flow cytometry analysis is shown: (**A**) CD14^+^CD163^−^ monocytes, (**B**) CD14^+^CD163^+^ monocytes, and (**C**) CD14^−^CD163^+^ monocytes. The mitochondrial membrane potential (Δφ) was analyzed using JC-1 staining followed by flow cytometric analysis on: (**D**) CD14^+^CD163^+^ monocytes, (**E**) CD14^+^CD163^+^ monocytes, and (**F**) CD14^+^CD163^−^ monocytes. Mean values ±SD are presented. Baseline, T4 h T8 h n = 9/group, T20 h n = 6/group. The statistical significance of differences between values at different timepoints for each group: ** *p* < 0.01, *** *p* < 0.001, **** *p* < 0.0001. SLA-DR swine leukocyte antigen-DR.

The expression of the major histocompatibility complex II (MHC II) on the surface of human monocytes is one of the best studied biomarkers of monocyte activation/deactivation in sepsis [21]. Therefore, we chose to analyze the changes in the expression of the porcine MHC II antigen—the swine leukocyte antigen SLA-DR—on the subsets of circulating monocytes. The expression of SLA-DR was the lowest on the CD14^+^CD163^−^ monocytes and it did not change significantly during endotoxemia regardless of the iNO + hydrocortisone treatment (Figure 3A). On the contrary, the high baseline expression of SLA-DR on CD14^+^CD163^+^ cells decreased twofold after 20 h of endotoxemic shock. Even though both groups reached similar levels of SLA-DR, the kinetics were more rapid in the control group in comparison to the treatment group (Figure 3B). The strongest reduction in SLA-DR expression occurred in the subpopulation of CD14^−^CD163^+^ monocytes, in which it was threefold on average without any differences between the groups (Figure 3C).

Additionally, we assessed the functionality of monocyte mitochondria using the JC-1 dye, which can be used to assess mitochondrial membrane potential (Δψ), the loss of which indicates early apoptosis [22]. Interestingly, no changes in Δψ were found in either the CD14^+^CD163^−^ or CD14^+^CD163^+^ cells after injecting LPS (Figure 3D,E). However, the Δψ of the CD14^−^CD163^+^ monocytes increased twofold 20 h after the induction of endotoxemia in both groups of animals (Figure 3F). The combined iNO + hydrocortisone treatment did not affect the Δψ in any of the studied monocyte subsets.

Together, these data show that endotoxemia decreases the antigen-presenting capacities of the circulating monocytes, while it does not significantly impair their mitochondrial function. Moreover, the evaluated dual treatment did not affect these functions of the circulating monocytes in endotoxemia.

### 3.4. Compartment-Specific Alterations in the Monocyte Functions in Swine Endotoxemia

**Figure 4:**
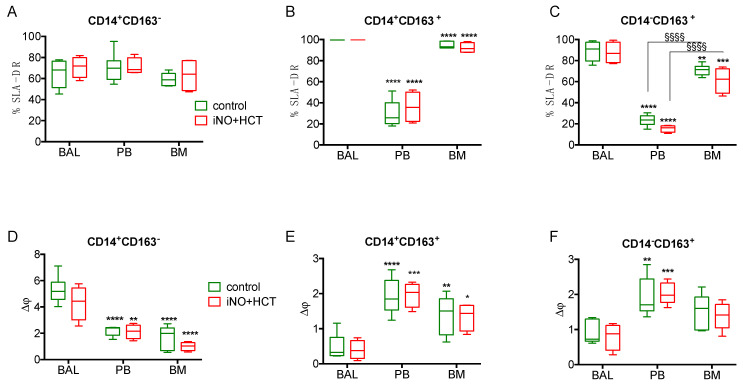
Compartment-specific changes in the activation status of monocytes following 20 h of endotoxemic shock. The expression of SLA-DR antigen was evaluated 20 h after the onset of endotoxemic shock in cells from the bronchoalveolar lavage fluid (BAL), peripheral blood (PB), and bone marrow (BM). The SLA-DR antigen was analyzed in: (**A**) CD14^+^CD163^−^ monocytes, (**B**) CD14^+^CD163^+^ monocytes, and (**C**) CD14^−^CD163^+^ monocytes. The mitochondria membrane potential (Δφ) was also analyzed in these three compartments in: (**D**) CD14^+^CD163^−^ monocytes, (**E**) CD14^+^CD163^+^ monocytes, and (**F**) CD14^−^CD163^+^ monocytes. Mean values ± SD are presented. n = 6/group. The statistical significance of differences between values for BAL vs. PB or BM in each experimental group: * *p* < 0.05, ** *p* < 0.01, *** *p* < 0.001, **** *p* < 0.0001. Significant differences between PB and BM §§§§ *p* < 0.0001. No differences were noted between control and treated group.

It has been suggested that the immune response during systemic inflammation in sepsis is compartmentalized, e.g., varies depending on the anatomical niche [2,23]. Therefore, we took advantage of this animal model study to analyze the impact of the combined treatment on the status of monocytes from endotoxemic pigs after the termination of the experiment from three compartments: peripheral blood, bone marrow, and lung (retrieved by bronchoalveolar lavage). The expression of SLA-DR antigen was similar on the CD14^+^CD163^−^ monocytes, regardless of the sampled site (Figure 4A). However, almost 100% of the CD14^+^CD163^+^ and CD14^−^CD163^+^ monocytes from the BALF expressed SLA-DR, and it was significantly higher in comparison to the peripheral blood and bone marrow counterparts (Figure 4B,C). The bone marrow CD14^−^CD163^+^ monocytes showed a significantly higher expression of SLA-DR in comparison to the peripheral blood cells regardless of the treatment (control: 63.8% vs. 23.3% *p* < 0.0001; treated: 61.2% vs. 15.4%, *p* < 0.0001, respectively; Figure 4C). The intercompartment differences between SLA-DR expression were not modulated by the treatment.

We also analyzed mitochondrial functionality in these compartments. The Δψ was significantly reduced in the peripheral blood and bone marrow CD14^+^CD163^−^ monocytes in comparison to the BALF-retrieved cells (Figure 4D). Surprisingly, the opposite relations were observed for the CD14^+^CD163^+^ and CD14^−^CD163^+^ monocytes, e.g., The Δψ of these populations were lowest in the BALF cells (Figure 4E,F). The combined iNO + hydrocortisone treatment did not affect the compartment-specific changes in the Δψ.

These findings support the hypothesis that the monocyte response in systemic endotoxemia is compartmentalized, and lung cells may be more resistant to the deactivation of their immune functions. Moreover, the iNO + hydrocortisone treatment does not influence the compartment-specific differences in monocyte activation status.

## 4. Discussion

We utilized a porcine model of prolonged resuscitated endotoxemic shock to investigate the effects of inhaled nitric oxide (iNO) and hydrocortisone (HCT) treatment on monocyte functions. Our study showed that the combined treatment has little effect on the circulating monocyte subsets and function as measured by the expression of the MHC II antigen and assessment of the mitochondrial membrane potential. We also gained insight into the compartmentalization of monocyte deactivation in endotoxemic shock and found that the iNO and HCT treatment does not affect it.

Large animal models of sepsis with intensive care-like treatment have great translational value, as they enable recapitulation of human-like treatment and the clinical-like measurement of physiological parameters [24]. Importantly, the physiological reactions of pigs share more similarities with humans than rodents. Additionally, experimental pigs are not specific-pathogen-free and they are exposed to natural microbiota and pathogens, making their immune reactions more human-like [24]. Here, we used our established model of 10 h of LPS infusion and 20 h of intensive care treatment that lead to the development of multi-organ failure but not the rapid death of the animals. Indeed, the presence of shock and organ failure in our model was evidenced by the rise in lactates and creatinine with a drop in cardiac output. However, shock resuscitation according to the Surviving Sepsis Guidelines [25] prevented a drop in arterial pressure and led to normalization of the lactate level. The design of our study involved starting the tested treatment 3 h after the onset of shock, which fulfills the translational requirements according to the current guidelines on the preclinical modeling of sepsis [4].

The combined iNO+HCT treatment helped to maintain a higher mean arterial pressure at 20 h from the onset of endotoxemia and to decrease the mean pulmonary arterial pressure, which confirms the beneficial effect of this combined treatment from our previous study [12]. It can be speculated that this circulatory effect is attributable mainly to HCT, as reported in some clinical studies [26]. In our previous porcine study, norepinephrine reduction was possible only when these two drugs were used in combination [12]. We also confirmed our previous report on the protective effects of this combined treatment on renal function [12].

The investigated treatment did not alter the kinetics of the white blood count nor the T cells during endotoxemia. Hydrocortisone treatment alone has been shown to affect circulating leucocytes in both human and porcine studies [27,28]. However, as our focus was on the monocytes, we combined two markers, CD14 and CD163, which has been shown to enable the identification of differential subsets of these cells. Analyzing the monocyte compartment in pigs is challenging, as it varies in different races, sexes, and ages [29]. Nevertheless, similarly to other reports, CD14^+^CD163^−^ and CD14^−^CD163^+^ monocytes were the major subtypes in the peripheral blood before the instillation of LPS [29,30]. CD14^+^CD163^−^ monocytes are considered developmentally early monocytes that resemble the human classical CD14^hi^ cells. In our model, we observed an insignificant increase in these cells during endotoxemia, which may have reflected the ongoing inflammatory response. At steady state, these cells presented the lowest expression of the MHC II antigen (SLA-DR), and interestingly, remained unaltered during endotoxemia. In turn, the CD14^−^CD163^+^ monocytes, which resemble human intermediate monocytes [19] and release the highest amount of TNF upon ex vivo stimulation [20], followed the kinetics of WBC and significantly decreased the expression of SLA-DR during endotoxemia. Interestingly, in our model, we observed an increase in CD14^+^CD163^+^ monocytes, which was much stronger in the control group than in the treatment group. Endotoxemia also reduced SLA-DR expression by this monocyte subset. In human sepsis, MHC II (HLA-DR) antigen expression is commonly used as a marker of immunosuppression, as it correlates with TNF production by the monocytes as well as the clinical course and development of secondary infections [31]. It can be hypothesized that the porcine endotoxemia model similarly induces immuno-reprogramming and a state of endotoxin tolerance of the blood monocytes; however, functional tests are required to support this. The occurrence of the phenomenon of endotoxin tolerance of blood cells has previously been reported in porcine endotoxemia studies [32,33]. The maximal endotoxin tolerance was shown to occur early after the instillation of LPS (6 h) [32], while we observed a steady drop in the expression of SLA-DR over time. A differential reduction in HLA-DR was observed in cardiothoracic surgery patients and was greatest in CD14^hi^ monocytes [34]; this is in contrast to our findings, although not all human studies confirmed this observation [35]. Of note, the combined treatment did not further decrease the expression of SLA-DR, which could have been expected given the immunosuppressive functions of hydrocortisone [34,36].

We also assessed the functional status of the monocyte mitochondrial membrane potential (Δφ) using the widely accepted JC-1 dye and flow cytometry technique [37]. Interestingly, only CD14^−^CD163^+^ monocytes displayed increased Δφ during endotoxemia, while in other populations, this parameter remained intact. This is an unexpected finding, as circulating monocytes from patients with septic shock had a decreased Δφ that correlated with the outcome [38]. The bioenergetic reprogramming of human monocytes in sepsis has also been shown by other methods and in other patients groups [39,40], and it is restored during recovery [41]. This clear discrepancy is difficult to understand; however, it could be model-dependent. As mentioned earlier, porcine monocytes can undergo endotoxin tolerance [33]. In a model of porcine polytrauma, Vollrath et al. showed that the functionality of monocytes is not unequivocally altered but rather time- and trigger- dependent [42], which shows the complexity of the monocyte response to trauma and probably sepsis.

In this study, we compared monocytes from the peripheral blood, lungs, and bone marrow and simultaneously assessed their status after 20 h of endotoxemia. The subpopulation of CD14^+^CD163^−^ expressed SLA-DR at a similar level regardless of localization. However, both CD14^+^CD163^+^ and CD14^−^CD163^+^ monocytes from the lungs preserved the SLA-DR expression, while peripheral blood monocytes were characterized by the greatest reduction. Bone marrow monocytes showed intermediate expression of SLA-DR. Inversely, the mitochondrial Δφ of CD14^+^CD163^−^ cells was highest in the lungs, while in both CD163^+^ monocyte subsets, it was highest in the peripheral blood. The results of a higher expression of SLA-DR in lung rather than blood monocytes is consistent with our previous findings from the analysis of BALF and blood monocytes from human septic shock patients [43]. The preserved SLA-DR expression in lung monocytes also supports the findings from a murine study that alveolar compartments prevent the development of endotoxin tolerance through the unique cytokine microenvironment [44]. As the changes in SLA-DR expression were opposite to the Δφ, this suggests that these two measures of monocyte functionality are differentially affected by endotoxemia. It also indicates that CD163^+^ monocytes differ from CD14^+^CD163^−^ cells in the response to endotoxemia. Our findings have important consequences for monitoring the status of monocytes in sepsis. They provide additional evidence that the status of circulating monocytes does not reflect tissue-resident cells and suggests caution with immunomodulatory treatment [45].

Our study had several limitations. The small group size is unfortunately typical in porcine studies due to the high workload, the costs of studying each animal, and the application of the 3R rules. Although animals were randomly assigned to either group, we were unable to perform a blinded study, due to the requirements for the iNO supply. An important limitation of this study was the lack of an ex vivo cytokine production functional test of the analyzed monocyte subsets. This aspect should be covered in future studies.

In conclusion, our study confirmed the kidney-protective action of the combined iNO + hydrocortisone treatment and did not show significant alterations in the monocyte subsets from this therapy. Our results expand the knowledge on porcine monocytes in a steady state and during endotoxemia. Importantly, we confirmed the compartmentalization of the monocyte response during endotoxemic shock, which highlights the importance of studying tissue-resident cells in addition to their circulating counterparts in sepsis.

### Clinical Implications

This study provided another line of evidence on the kidney-protective effects of the combined treatment with iNO and hydrocortisone in the translational model of porcine endotoxemic shock. Endotoxemia induced a reduction in the antigen-presenting capacity of the circulating monocytes comparable to human sepsis, and the potentially immunosuppressive iNO and hydrocortisone treatment did not further decrease it. The studied treatment also did not affect the preserved functionality of lung monocytes. Taken together, our results obtained from a relevant large animal model suggest that the combined use of inhaled nitric oxide and hydrocortisone in septic shock can be safe and beneficial, but appropriate clinical trials should be performed before any clinical statements can be made.

## Data Availability

The raw data supporting the conclusions of this manuscript will be made available by the authors, without undue reservation, to any qualified researcher.

## Supplementary Materials

The following supporting information can be downloaded at: https://www.mdpi.com/article/10.3390/jcm11092641/s1, Table S1: The ARRIVE guidelines 2.0: author checklist.
